# Quercetin prevents rhinovirus-induced progression of lung disease in mice with COPD phenotype

**DOI:** 10.1371/journal.pone.0199612

**Published:** 2018-07-05

**Authors:** Mohammad Farazuddin, Rahul Mishra, Yaxun Jing, Vikram Srivastava, Adam T. Comstock, Umadevi S. Sajjan

**Affiliations:** 1 Department of Pediatrics and Communicable Diseases, University of Michigan, Ann Arbor, Michigan, United States of America; 2 Department of Thoracic Surgery and Medicine, Temple University, Philadelphia, Pennsylvania, United States of America; 3 Department of Physiology, Temple University, Philadelphia, Pennsylvania, United States of America; Centre National de la Recherche Scientifique, FRANCE

## Abstract

Acute exacerbations are the major cause of morbidity and mortality in patients with chronic obstructive pulmonary disease (COPD). Rhinovirus, which causes acute exacerbations may also accelerate progression of lung disease in these patients. Current therapies reduces the respiratory symptoms and does not treat the root cause of exacerbations effectively. We hypothesized that quercetin, a potent antioxidant and anti-inflammatory agent with antiviral properties may be useful in treating rhinovirus-induced changes in COPD. Mice with COPD phenotype maintained on control or quercetin diet and normal mice were infected with sham or rhinovirus, and after 14 days mice were examined for changes in lung mechanics and lung inflammation. Rhinovirus-infected normal mice showed no changes in lung mechanics or histology. In contrast, rhinovirus-infected mice with COPD phenotype showed reduction in elastic recoiling and increase in lung inflammation, goblet cell metaplasia, and airways cholinergic responsiveness compared to sham-infected mice. Interestingly, rhinovirus-infected mice with COPD phenotype also showed accumulation of neutrophils, CD11b^+^/CD11c^+^ macrophages and CD8+ T cells in the lungs. Quercetin supplementation attenuated rhinovirus-induced all the pathologic changes in mice with COPD phenotype. Together these results indicate that quercetin effectively mitigates rhinovirus-induced progression of lung disease in a mouse model of COPD. Therefore, quercetin may be beneficial in the treatment of rhinovirus-associated exacerbations and preventing progression of lung disease in COPD.

## Introduction

Chronic obstructive pulmonary disease (COPD), a relatively prevalent lung disease is also one of the leading causes of morbidity and mortality worldwide [1]. Acute exacerbations of COPD are the major cause of morbidity and mortality because, it is often associated with hospitalizations, accelerated loss of lung function [2], and decreased quality of life [3,4].

Respiratory infections are responsible for 50 to 70% of COPD exacerbations and approximately 1/3^rd^ to 1/2 of these were associated with virus infections with rhinovirus (RV) being the most commonly detected virus [5,6]. RV predominantly causes self-limiting upper respiratory illness with no significant involvement of lower respiratory tract in healthy individuals. Accumulating clinical evidence indicate casual relationship between RV infection and worsening of both upper and lower respiratory symptoms, and development of secondary bacterial infections in COPD patients [5,7]. Additionally, frequent and severe exacerbations were likely to be associated with RV infections [7]. An elegant study involving experimental infection of COPD or normal subjects patients with RV provided direct evidence demonstrating that RV induces respiratory illness which is more severe and prolonged in COPD than in normal [8]. Further RV-infected COPD patients showed persistent expression of inflammatory cytokines, enhanced oxidative stress and accumulation of inflammatory cells in the lungs [8–10]. The prolonged responses were associated with relatively longer persistence and higher load of virus. Inhaled corticosteroids, which are currently being used to treat exacerbations, modestly reduce respiratory symptoms and sometimes increases the risk for secondary bacterial infections [11–13]. Therefore, better therapies with relatively no side effects are needed for treatment of COPD exacerbations associated with rhinovirus infections.

Quercetin is a plant polyphenol and has potent antioxidant and anti-inflammatory properties. Previous epidemiological studies have suggested that consumption of quercetin-rich diet decreases the risk for development of asthma [14], bronchial hyper-reactivity [14] and chronic obstructive pulmonary disease [15]. Previously, we demonstrated that oral treatment with quercetin prevents further deterioration of lung function and also significantly reduces goblet cell metaplasia and lung inflammation in a mice displaying COPD-like features [16]. Additionally, we also showed that quercetin inhibits RV replication in airway epithelial cells *in vitro* and in a mouse model of RV infection *in vivo* [17]. Therefore, we hypothesized that quercetin supplementation may augment viral clearance and mitigate RV-induced pathologic changes in a mouse model of COPD.

Previously we demonstrated that mice exposed to combination of cigarette smoke and heat-killed non-typeable *H*. *influenzae* (NTHi) show mild to moderate emphysema, diffuse lung inflammation encompassing both conductive airways and parenchyma, and goblet cell metaplasia [18]. Following RV infection, unlike room air-exposed mice, mice exposed to combination of cigarette smoke and heat-killed NTHi (mice with COPD phenotype) showed persistence of virus up to 4 days post infection, enhanced lung inflammation and goblet cell metaplasia, increased expression of mucin genes and pro-inflammatory cytokines similar to that observed in RV-infected COPD patients. In contrast, mice exposed to heat-killed NTHi or cigarette smoke alone clear virus similar to room air-exposed mice and does not induce significant changes in lung inflammation or cytokine expression following RV infection. Based on these observations, we choose to use mice exposed to both cigarette smoke and heat-killed NTHi which display mild COPD phenotype to examine the long-term effects of rhinovirus. Since, we have already demonstrated that quercetin reduces lung inflammation and improves lung mechanics in mice displaying COPD-like features [16], and augments viral clearance and reduces RV-induced acute lung inflammation in normal mice [17], the present study was focused on examining the effects of quercetin in mitigating RV-induced long-term effects in mice with COPD phenotype.

RV16, which binds to human ICAM-1 does not infect mouse airways because of species specificity. In contrast, RV1B, which binds to a family of low density lipoprotein receptors infect mouse epithelial cells *in vitro* and also mouse airways *in vivo* [19,20]. Additionally, RV1B stimulates similar cytokine responses as RV16 in human airway epithelial cells. Therefore, RV1B, which is capable of infecting mouse airways was used in the present study.

## Materials and methods

### Mice

**Fig 1:**
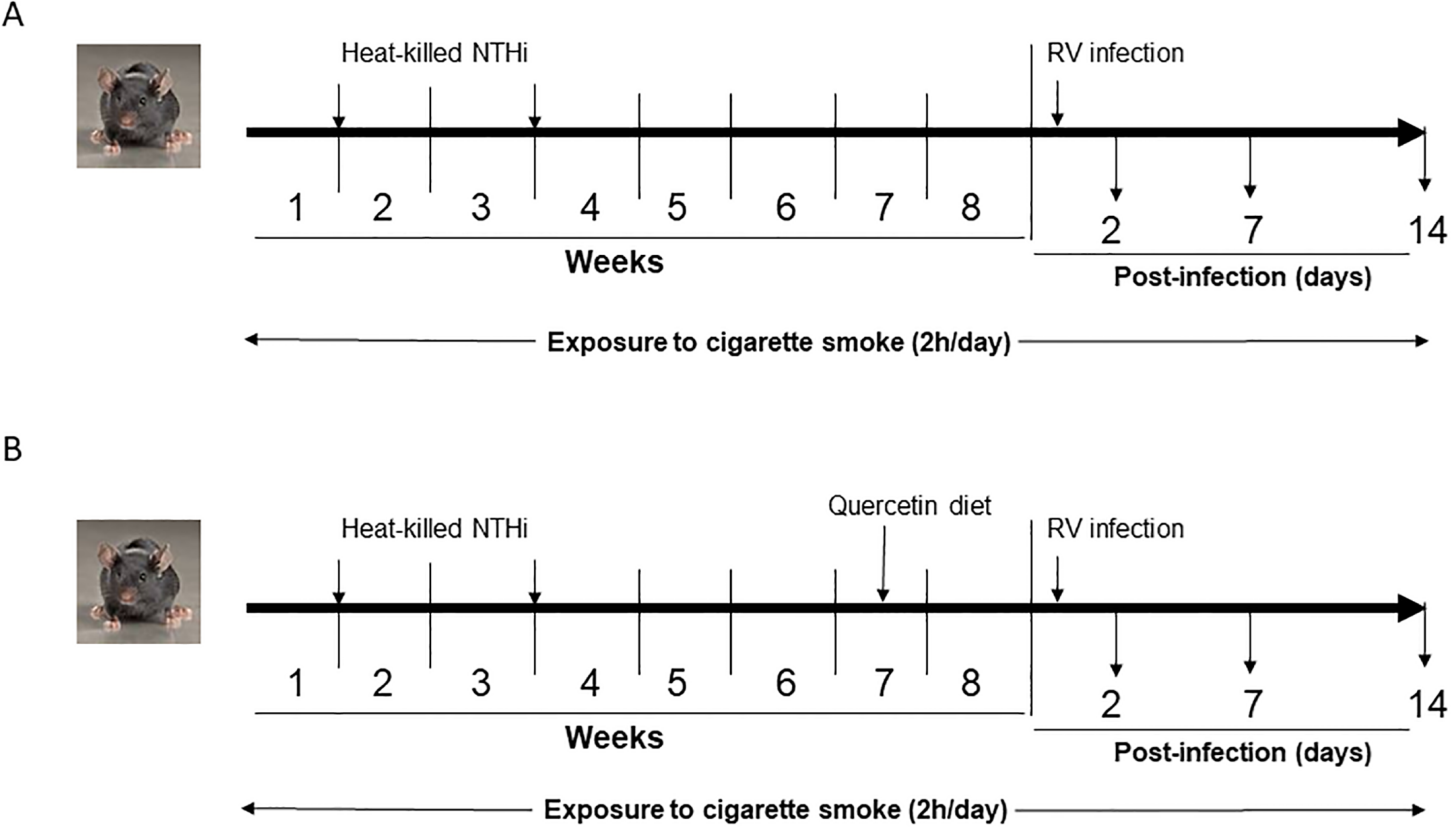
Scheme showing exposure of mice to cigarette smoke and heat-killed NTHi, treatment with RV infection and maintenance of mice on quercetin diet.

Mild COPD-like lung disease was induced in C57BL/6 mice as described previously [21]. Briefly, 6–8 weeks old female mice were subjected to whole body cigarette smoke exposure for 2h a day, 5 days a week for 8 weeks using TE10B cigarette smoking machine and standardized 3R4F research cigarettes. Total suspended particulates (TSP) level in the exposure chamber was maintained at 150 mg/m^3^ throughout the experiment. Mice were also treated with heat-killed NTHi (equivalent to 5 x 10^6^ CFU) on days 7 and 21 by intranasal route (Fig 1A). We refer to mice exposed to combination of cigarette smoke and heat-killed NTHi as mice with COPD phenotype throughout this report. Mice exposed to room air were used as controls and here we refer to them normal mice. At the end of experiments, mice were sacrificed by asphyxiation. All the experimental procedures were approved by the Institutional Animal Care and Use Committee of the University of Michigan, Ann Arbor and Temple University, Philadelphia. To determine the minimum number of animals required to get significant difference between the infected and uninfected groups was calculated by power analysis using the data from our previous study [21] in which we examined the effect of RV on the expression of Muc5AC in mice with COPD phenotype. With the calculated effect size of 2.45, α = 0.05 and power of 0.95, we determined that we will require a minimum of 5 animals per group. Therefore, we used 6 animals per group in most of the experiments to achieve significance.

### Rhinovirus and infection

Both RV1B and H1 HeLa cells were purchased from American Type Culture Collection, Manassas, VA. Stocks of RV1B were prepared by infecting H1HeLa cells with RV1B and subjecting HeLa cell supernatants to ultrafiltration as described previously [22]. Similarly concentrated and purified cell supernatants from uninfected H1HeLa cells was used as sham controls. Normal mice and mice with COPD phenotype were infected with RV (50 ul of PBS containing 5 x 10^6^ PFU) or an equal volume of sham preparation by intranasal route as described previously [23]. Exposure of mice with COPD phenotype to cigarette smoke was continued until sacrificed. Mice were sacrificed at 2, 7, or 14 days post-infection by asphyxiation.

### Quercetin supplementation

Mice with COPD phenotype were shifted to control or 0.1% quercetin containing diet (Bio-Serve, Flemington, NJ) 10 days prior to RV infection and maintained on the same diet until the mice were sacrificed (Fig 1B). Quercetin was kindly provided by Quercegen Pharmaceuticals, Boston, MA and was 99.4% pure by HPLC and the custom diet containing 100 mg/kg and control diet was prepared and supplied by BioServ, Flemington, NJ. Blood levels of quercetin at the end of an experiment was 0.3290 ± 0.091 μM and 0.0094 ± 0.0001 in mice fed with quercetin diet and control diet respectively.

### RNA isolation and qPCR

After relevant treatment, lungs were collected under aseptic conditions and homogenized in 2 ml PBS. An aliquot of lung homogenate was immediately mixed with TRIZOL, total RNA was purified using RNeasy miRNA kit (Qiagen, Alameda, CA), and cDNA was synthesized using Taqman reverse transcription kit (Applied Biosystems Life Technologies, Carlsbad, CA). cDNA was then used to determine the mRNA expression of CXCL-1, CXCL-10, CCL2, CCL3, IL-17, IFN-α, IFN-β, IFN-λ_2_, TNF-α, IFN-γ, Muc5ac, Gob5 and β-actin (house-keeping gene) by quantitative qPCR using gene specific primers and probes purchased from either Thermo Fisher Scientific (Waltham, MA) or Integrated DNA Technologies (Coralville, IA). To detect viral RNA, total RNA isolated from the lungs was subjected to quantitative Taqman qPCR as described previously [19] and expressed as number of vRNA copies per 10 μg of total RNA.

### ELISA

Supernatants from lung homogenates were used for ELISA to quantify protein levels of cytokines. All ELISA kits were purchased from R & D systems (Minneapolis, MN).

### Differential cell counts and flow cytometry

Lungs were perfused with cold PBS via right ventricle, minced and digested with collagenase IV (5 mg/ml) and DNase I [24]. Lung digests were passed through 70 μ filter, cells were harvested by centrifugation, and treated with RBC lysis buffer. Cells were then washed and suspended in PBS.

To determine differential cell counts, an aliquot of cell suspension equivalent to 1 x 10^6^ cells was labeled with antimouse CD45 antibody conjugated with magnetic microbeads (Miltenyi Biotec Inc, Auburn, CA) to isolate CD45 positive cells. Cytospins of CD45 positive cells were prepared, stained with DiffQucik and number of macrophages, neutrophils and T cells were determined.

For flow cytometry analysis, cells obtained after RBC lysis were incubated with Zombie UV^™^ (BioLegend) to label dead cells and stained with fluorescence-labeled Abs against surface markers of leukocytes, such as CD45, CD11c, CD11b, F4/80, CD3e, CD8 and CD4. Appropriate isotype-matched controls and fluorescence minus one (FMO) were used in all experiments. All antibodies were purchased from BioLegend (San Diego, CA). Cells were fixed and analyzed in BD LSR II Flow cytometerI (BD Biosciences) and data was analyzed using FlowJO version 10 (Tree Star, Ashland, OR).

### Histology

Lungs were inflation fixed at a constant pressure of 30 cm.H_2_O for 30 min. Lungs were embedded in paraffin, and 5 μ thick sagittal sections were stained with hematoxylin and eosin (H & E) to assess histology or periodic acid-Schiff (PAS) to detect goblet cells. H & E-stained sections were scored by an experienced laboratory personnel, who was blinded for treatment using a semiquantitative scale in the range of 0 to 5 [25]. Zero on this scale indicated no inflammatory change, while 5 represented severe inflammation. Number of PAS-positive cells per 100 μ of airway epithelium was counted to quantify goblet cells as described [21,23]. Chord length was determined as described previously [23].

### Analysis of lung mechanics and airway resistance

Dynamic lung elastance and compliance, and pressure-volume relationship were measured as described previously using a miniature computerized flexivent ventilator (Scireq, Canada) [23]. Airway responsiveness to nebulized methacholine was measured as described previously using Buxco FinePointe operating system connected to mechanical ventillator (Wilmington, NC). [23].

### Statistical analysis

Results are expressed as mean ± SEM or median with range of data. Data were analyzed by using SigmaStat statistical software (Systat Software, San Jose, CA). For the normally distributed data, we used ANOVA with Tukey’s post-hoc analysis or 2-way ANOVA. If the data were not normally distributed, it was analyzed by non-parametric test, ANOVA on ranks with Kruskal-Wallis H test. A p value ≤ 0.05 was considered significant.

## Results

### Quercetin reduces rhinovirus-induced persistent lung inflammation in mice with COPD phenotype

**Fig 2:**
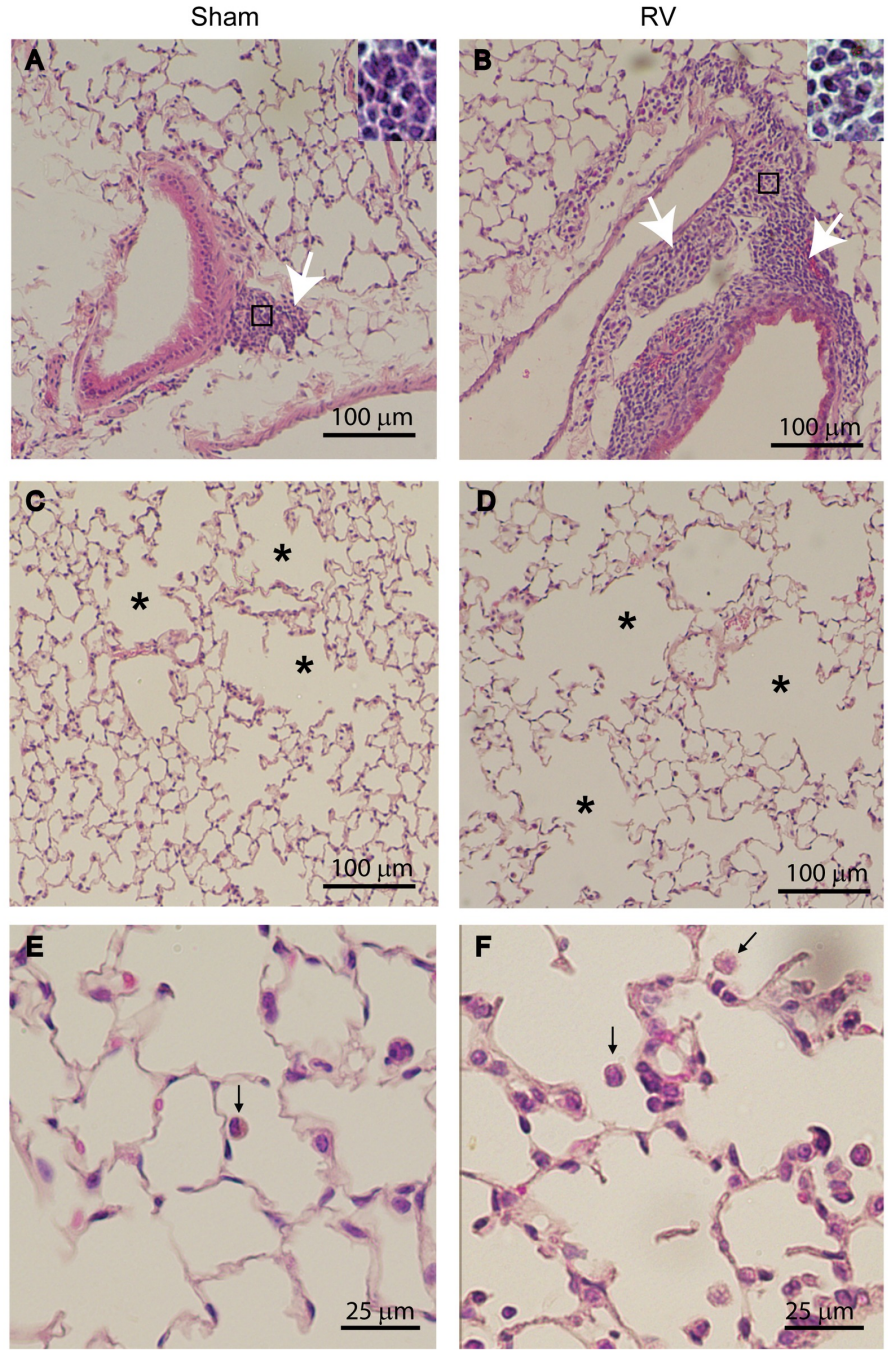
Mice with COPD phenotype show persistent inflammation after RV infection. H & E-stained lung sections from sham or RV-infected mice with COPD phenotype illustrate that compared to sham-, RV-infected mice show (A and B) increased inflammation in peribronchiolar and perivascular areas in COPD mice (white arrows), and (C and D) enlarged air spaces in lung parenchyma (asterisks). (E and F), represent higher magnification of parenchyma showing macrophages in the air space (represented by black arrow). Insets in (A and B) represent magnified area marked in rectangle showing predominantly mononuclear inflammatory cells. Images are representative of 6 mice per group from two independent experiments.

**Fig 3:**
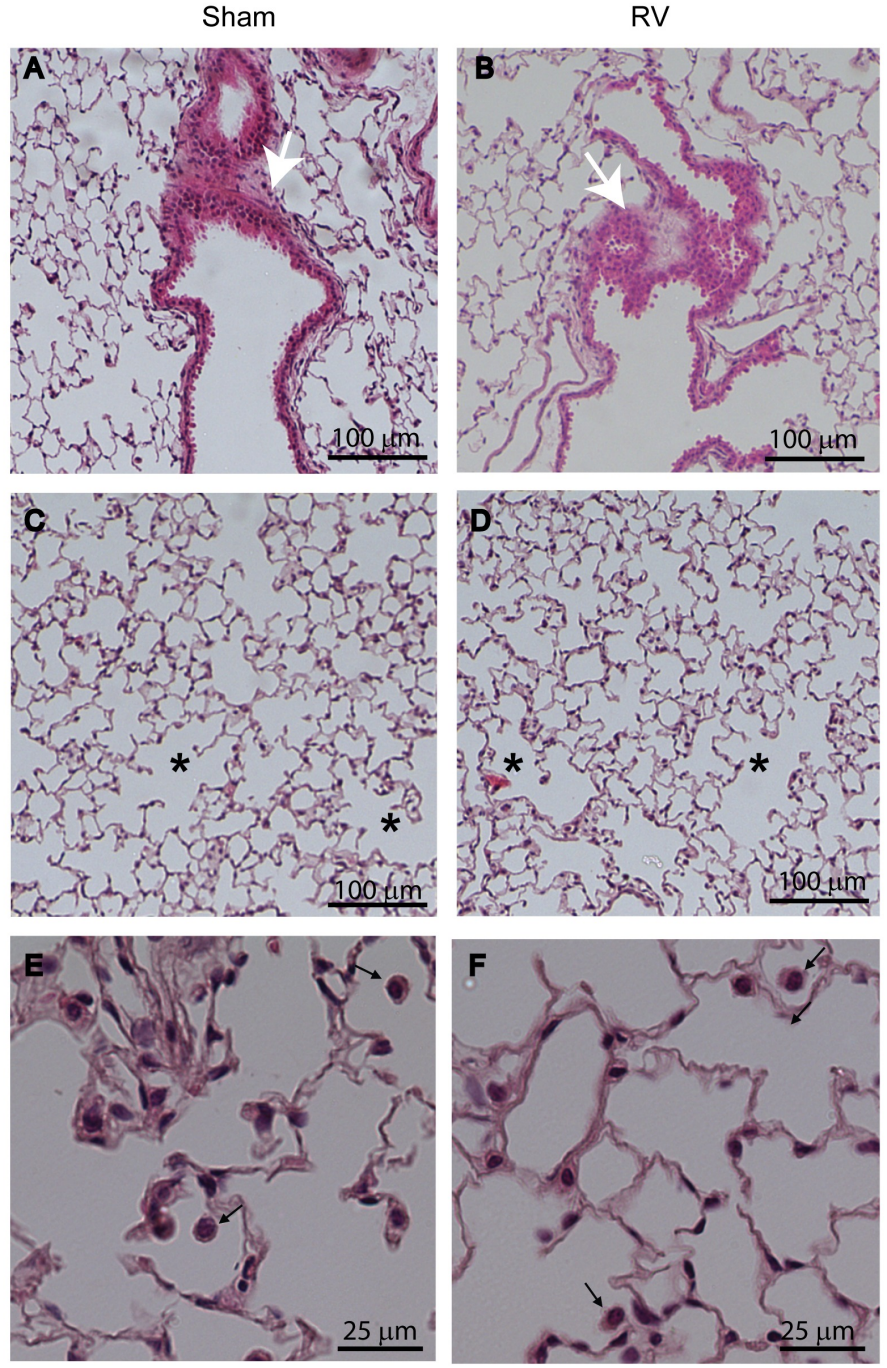
Quercetin blocks RV-induced inflammation in mice with COPD phenotype. H & E-stained lung sections from RV-infected mice with COPD phenotype maintained on quercetin containing diet show (A and B), reduced peribronchial inflammation (white arrows) and (C and D) no enlargement in air space (asterisk). (E and F), represent higher magnifcation of parenchyma showing macrophages in the air space (represented by black arrow). Images are representative of 6 mice per group.

Previously, we demonstrated that compared to RV-infected normal mice, mice with COPD phenotype show increased lung inflammation up to 4 days following RV infection. To examine whether mice with COPD phenotype resolves lung inflammation induced by RV, we examined lung histology at 14 days post-RV infection. Irrespective of infection, normal mice showed no histological changes (S1 Fig). Mice with COPD phenotype infected with RV and maintained on control diet showed mild to moderate peribronchial and perivascular inflammation that is somewhat exaggerated compared to sham-infected mice (Fig 2A and 2B). Additionally, these mice also showed enhanced emphysematous changes with more inflammatory cells (Fig 2C to 2F). In contrast, COPD mice maintained on quercetin-containing diet and then infected with RV showed substantially less lung inflammation and emphysematous changes (Fig 3A to 3F) than mice fed with normal diet (Fig 2A to 2F). Semi-quantitation of lung inflammation revealed the scores in the range of 3–4 in RV-infected COPD mice maintained on control diet and 1–2 for mice maintained on quercetin diet (Table 1).

**Table 1:** Histopathological scores of RV-infected COPD mice maintained on control or quercetin diet.

Phenotype and Infection	Diet	Score^a^		
		Peribronchial and perivascular inflammation	Alevolar inflammation	emphysema
COPD mice- sham	Control	1	1	2
		2	1	1
		1	1	2
		1	1	2
		2	2	1
		3	1	2
COPD mice -RV	Control	3	2	3
		4	1	4
		2	2	2
		3	3	4
		3	2	3
		4	3	3
COPD mice—sham	Quercetin	2	1	2
		1	1	2
		0	2	1
		1	1	2
		0	0	1
		2	1	2
COPD mice—RV	Quercetin	2	1	1
		1	0	2
		1	1	2
		2	0	2
		1	1	1
		0	1	1

‘^a^’ represent score for each mouse

To determine whether the sustained lung inflammation in RV-infected mice with COPD phenotype parallels with viral persistence, we determined viral load by measuring viral RNA. We used this method because it is more sensitive than plaque assay that detects infectious virus. Additionally, persistence of viral RNA in the absence of infectious virus may be sufficient to induce lung inflammation. At 2 and 4 days post-infection both normal and mice with COPD phenotype showed viral RNA (S2 Fig). At 7 days post-infection while only 2 out of 6 normal mice had detectable levels of viral RNA, 5 out of 6 mice with COPD phenotype showed viral RNA albeit at very low levels. At 10 days post-infection neither normal nor mice with COPD phenotype had detectable levels of viral RNA in their lungs (data not shown). Mice with COPD phenotype on quercetin diet showed 1 to 2 logs less viral RNA than mice on control diet at all time points. These results indicate that lung inflammation persists even in the absence of detectable viral RNA and that quercetin enhances viral clearance in addition to ameliorating lung inflammation in mice with COPD phenotype.

### RV causes sustained increase in the expression of inflammatory cytokines

**Fig 4:**
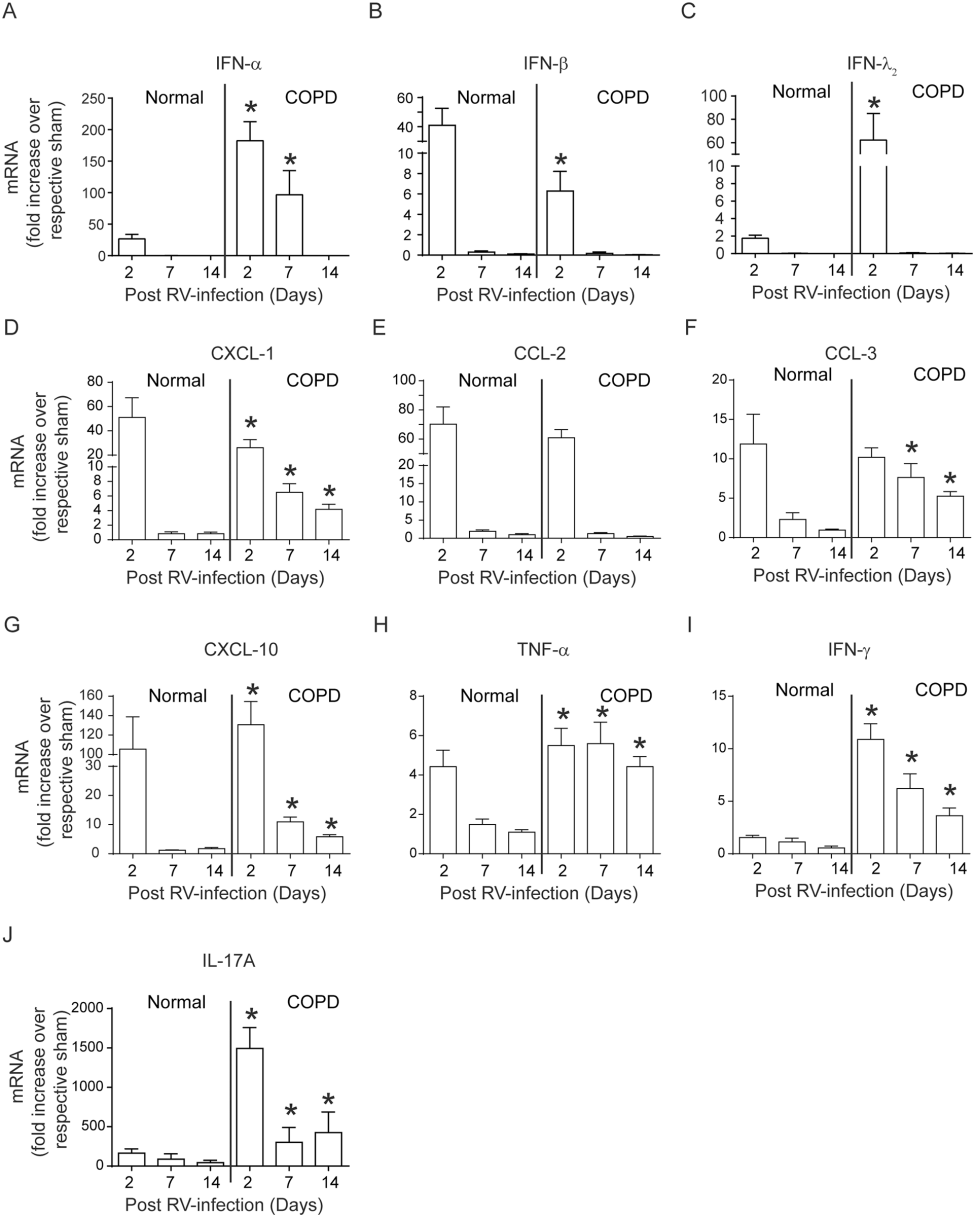
Mice with COPD phenotype show sustained expression of cytokines following RV infection. Total RNA isolated from the lungs of normal mice and mice with COPD phenotype at 2, 7 and 14 days post-infection was used to determine the mRNA expression of cytokines by qPCR. Data was normalized to house keeping gene, β-actin and expressed as fold expression over respective sham-infected animals. Data represent mean ± SEM calculated from two independent experiments with a total of 6 mice per group (*p≤0.05, different from normal mice at respective time point; ANOVA with Tukey post-hoc test).

**Fig 5:**
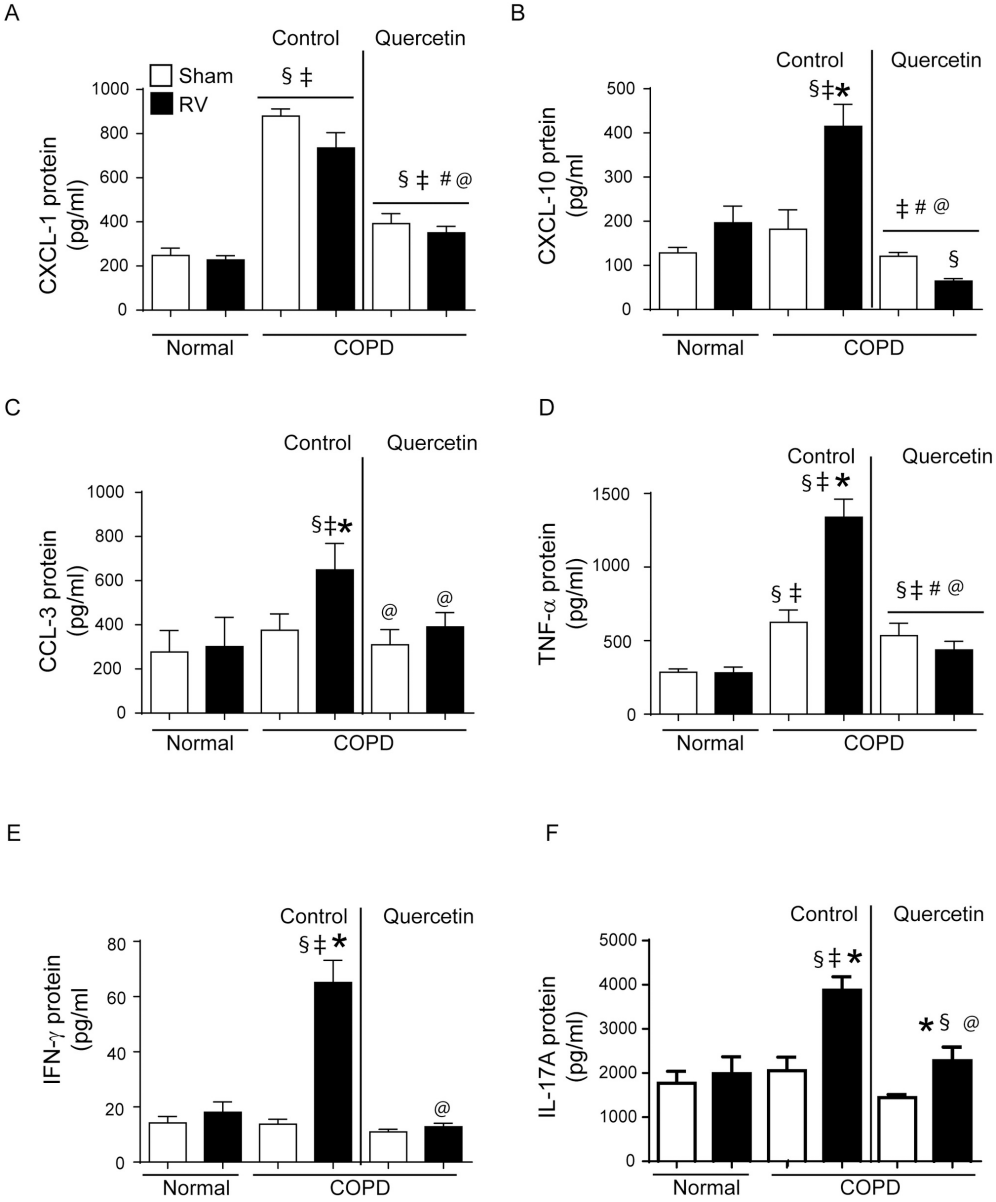
Querecetin blocks RV-induced sustained increase in cytokines at protein level. Supernatants from lung homogenates of sham or RV infected normal and COPD mice were used for detection of cytokines by ELISA. Data represent mean ± SEM calculated from two independent experiments with a total of 6 mice per group (* p≤0.05, different from respective sham; § p≤0.05, different from normal sham; ‡ p≤0.05, different from normal RV; # p≤0.05, different from sham-infected COPD mice on control diet; @ p≤0.05, different from RV-infected COPD mice on control diet ANOVA with Tukey post-hoc test).

The mRNA expression of selected cytokines that are involved in antiviral responses, IFN-α, IFN-β, IFN- λ_2_, neutrophil and macrophage chemoattractants, CXCL-1, CCL2, macrophage-derived inflammatory mediator, CCL3, T cell chemoattractant, CXCL-10 and T cell derived cytokines, TNF-α, IFN-γ and IL-17A was determined at 2, 7, and 14 days post RV-infection. Both normal mice and mice with COPD phenotype showed increase in the expression of all the cytokines at 2 days post-infection compared to respective sham-infected animals, which returned to basal levels in normal mice by day 7 post-infection (Fig 4A to 4J). Although mRNA levels of all these cytokines reduced in RV-infected mice with COPD phenotype, the levels of CXCL-1, CXCL-10, CCL3, TNF-α, IL-17A and IFN-γ remained high up to 14 days post-infection. However at protein level, only CCL3, CXCL-10, IL-17, TNF-α and IFN-γ were found to be higher in RV-infected mice with COPD phenotype compared to respective sham (Fig 5A to 5F). On the other hand, mice maintained on quercetin diet did not show increase in the protein or mRNA levels of CXCL-1, CXCL-10, IL-17, CCL3, TNF-α and IFN-γ (Fig 5A to 5F and S3 Fig).

### RV promotes accumulation of neutrophils, macrophages and T cells in the lungs of mice with COPD phenotype

**Fig 6:**
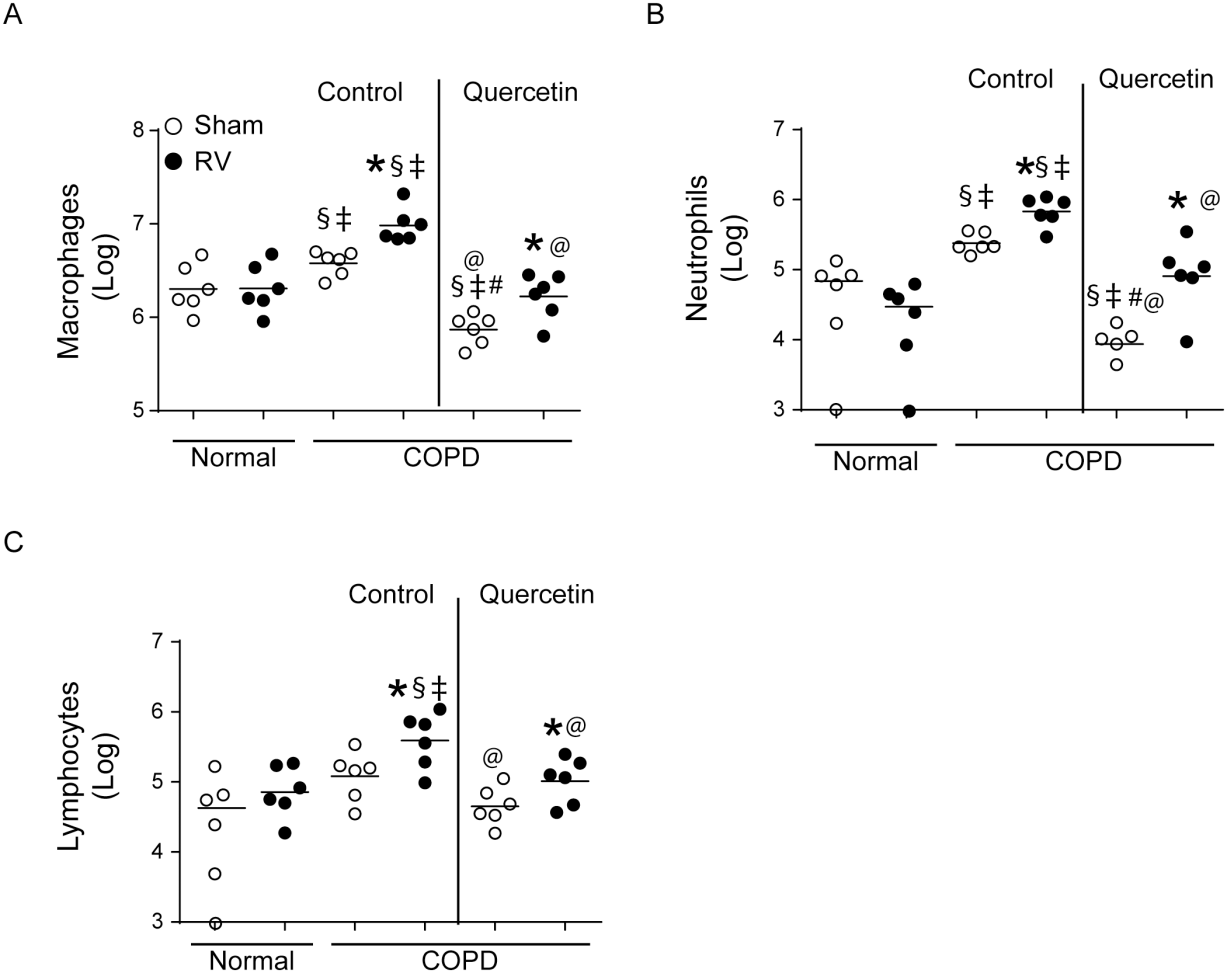
Quercetin reduces accumulation of inflammatory cells in RV-infected mice with COPD phenotype. Cytospins of CD45+ cells isolated from lung digests of sham- or RV-infected mice were stained with DiffQuick and different cell types were counted under microscope. Experiment was performed twice with 3 mice per group. Data represent median and range (* p≤0.05, different from respective sham; § p≤0.05, different from normal sham; ‡ p≤0.05, different from normal RV; # p≤0.05, different from sham-infected COPD mice on control diet; @ p≤0.05, different from RV-infected COPD mice on control diet ANOVA on ranks with Kruskal-Wallis H test).

In order to determine what type of inflammatory cells accumulate in the lungs of mice with COPD phenotype following RV infection we assessed differential cell counts in lung homogenates obtained at 14 days post-RV infection. Mice with COPD phenotype showed more neutrophils, macrophages and T cells as previously observed (Fig 6A–6C). Following RV infection normal mice showed slightly increased T cells, but not neutrophils or macrophages. In contrast, RV-infected mice with COPD phenotype, showed further increases in neutrophils, T cells and macrophages correlating with persistent increase in cytokine levels. Mice maintained on quercetin diet showed reduction in all three cell types corroborating with reduced levels of chemokines.

**Fig 7:**
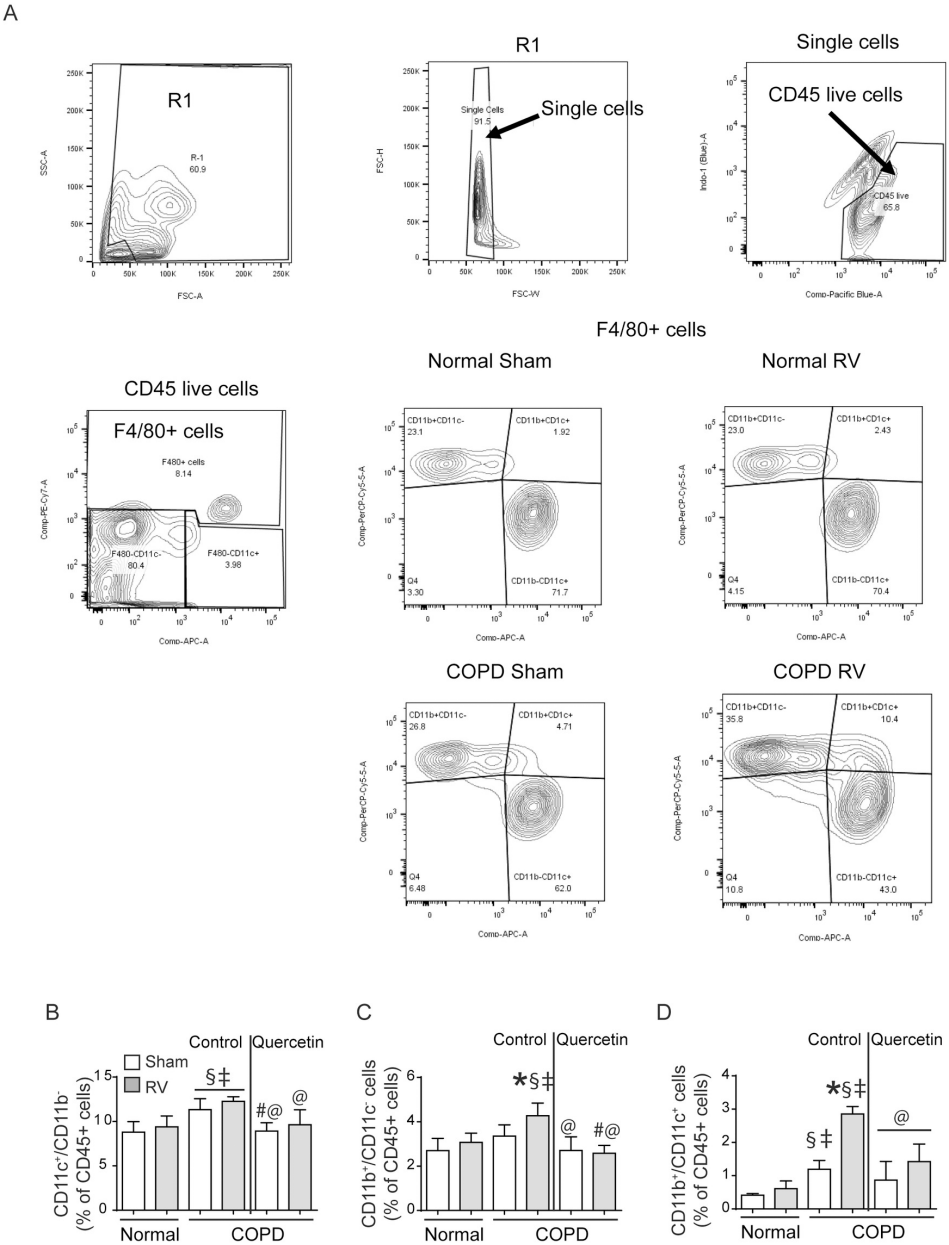
RV-infected COPD mice show accumulation of intermediate macrophages. Single cell suspensions from lung digest were stained with antibodies to CD45, F4/80, CD11b and CD11c to detect subtypes of macrophages. (A) illustrates gating strategy used for detection of subtypes of macrophages, (B to D) quantification of CD11c+ alveolar macrophages, CD11b+ interstitial macrophages and CD11b+/CD11c+ intermediate macrophages respectively. Data represent mean ± SEM calculated from two independent experiments with a total of 6 mice per group (* p≤0.05, different from respective sham; § p≤0.05, different from normal sham; ‡ p≤0.05, different from normal RV; @ p≤0.05, different from RV-infected COPD mice on control diet; ANOVA with Tukey post-hoc test).

Next, we conducted flow cytometry to determine subpopulation of accumulated macrophages and T cells. Analysis of macrophage population indicated three different types of macrophages/monocytes CD11b^+^/CDllc^-^ (lung monocytes), CD11c^+^/ CD11b^+^ (intermediate macrophages) and CD11c^+^/CD11b^-^ (alveolar macrophages) (Fig 7A). No significant differences were observed in any of the macrophage population between sham- and RV-infected normal mice (Fig 7B to 7D). Compared to normal, COPD mice showed small increases in alveolar and intermediate macrophage population. Intermediate, but not alveolar macrophage population significantly increased following RV infection in mice with COPD phenotype. Lung monocyte population also increased following RV infection in mice with COPD phenotype, but to a smaller extent. Quercetin treatment significantly reduced both alveolar and intermediate macrophage population, and also lung monocyte population in RV-infected mice with COPD phenotype.

**Fig 8:**
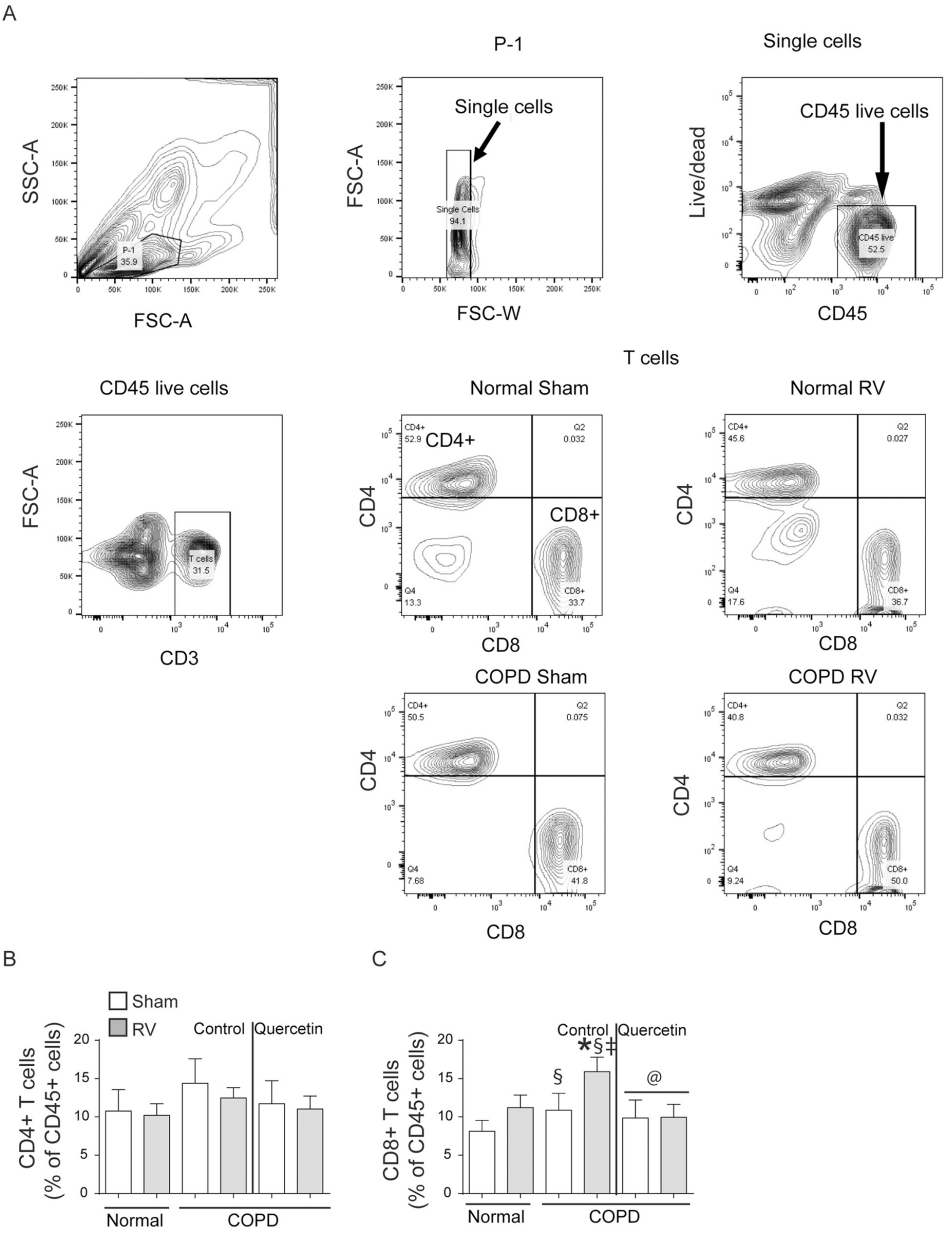
RV-infected COPD mice show increase in CD8+ T cell population. Single cell suspensions from lung digest were stained with antibodies to CD45, CD3, CD8 and CD4 to detect subtypes of T cells. (A) illustrates gating strategy used for detection of subtypes of T cells, (B and C) quantification of CD4+ and CD8+ T cells. Data represent mean ± SEM calculated from two independent experiments with a total of 6 mice per group (* p≤0.05, different from respective sham; § p≤0.05, different from normal sham; ‡ p≤0.05, different from normal RV; @ p≤0.05, different from RV-infected COPD mice on control diet; ANOVA with Tukey post-hoc test).

Gating strategy for subtypes of T cells is shown in Fig 8A. There was no difference in total CD4+ T cells between normal and mice with COPD phenotype (Fig 8B). Both normal and mice with COPD phenotype infected with RV showed significant increase CD8+ cells, but the increase was much higher in mice with COPD phenotype (Fig 8C). Quercetin treatment significantly reduced accumulation of CD8+ T cell population in RV-infected mice with COPD phenotype.

### Quercetin inhibits RV-induced mucus metaplasia in mice with COPD phenotype

**Fig 9:**
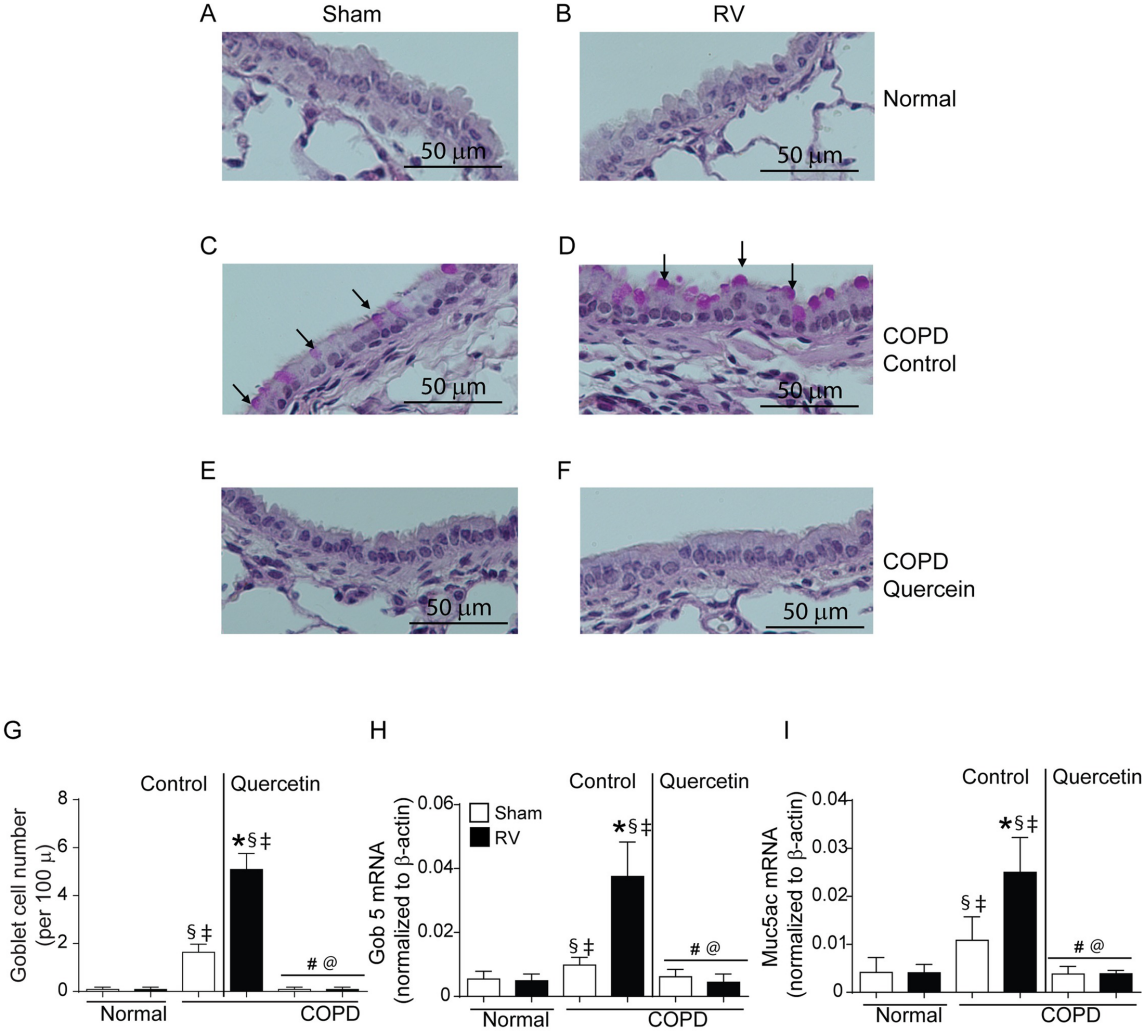
RV-infected mice with COPD phenotype show enhanced goblet cells metaplasia. Five micron thick paraffin sections were deparaffinized and stained with PAS to visualize goblet cells (arrows). (A and B) normal mice (C and D) mice with COPD phenotype maintained on control diet, (E and F) mice with COPD phenotype maintained quercetin diet. Images are representative of 6 mice per group. (F) quantitation indicate significantly more goblet cells RV-infected mice with COPD phenotype. (G and H) expression of Gob5 and Muc5ac was determined by qPCR using total lung RNA isolated from RV- or sham-infected normal and mice with COPD phenotype and data normalized to β-actin and expressed as fold change over respective sham-infected animals. Data in (G to I) represent mean ± SEM (* p≤0.05, different from respective sham; § p≤0.05, different from normal sham; ‡ p≤0.05, different from normal RV; # p≤0.05, different from sham-infected COPD mice on control diet; @ p≤0.05, different from RV-infected COPD mice on control diet, ANOVA with Tukey post-hoc test).

Assessment of PAS-stained sections indicated that compared to normal, mice with COPD phenotype show more goblet cells in small airways as observed previously (Fig 9A and 9C) [21]. Fourteen days after RV infection, while mice with COPD mice maintained on control diet showed further increase in goblet cells (Fig 9B and 9D), mice maintained on quercetin diet showed no goblet cells irrespective of infection and looked similar to normal mice (Fig 9E and 9F). Quantitation of goblet cells indicated significant increase in the number of goblet cells in COPD mice compared to normal mice, which further increased following RV infection and treatment with quercetin completely reduced the number of goblet cells in these mice (Fig 9G). Increase in goblet cells in mice with COPD phenotype prior to and after RV infection was accompanied with increased mRNA expression of goblet cell markers Gob5 and mucin gene Muc5AC (Fig 9H and 9I). Mice maintained on quercetin diet inhibited not only RV-induced expression of Gob5 and mucin genes, but also at basal levels in mice with COPD phenotype.

### Quercetin prevents progression of RV-induced emphysematous changes in mice with COPD phenotype

**Fig 10:**
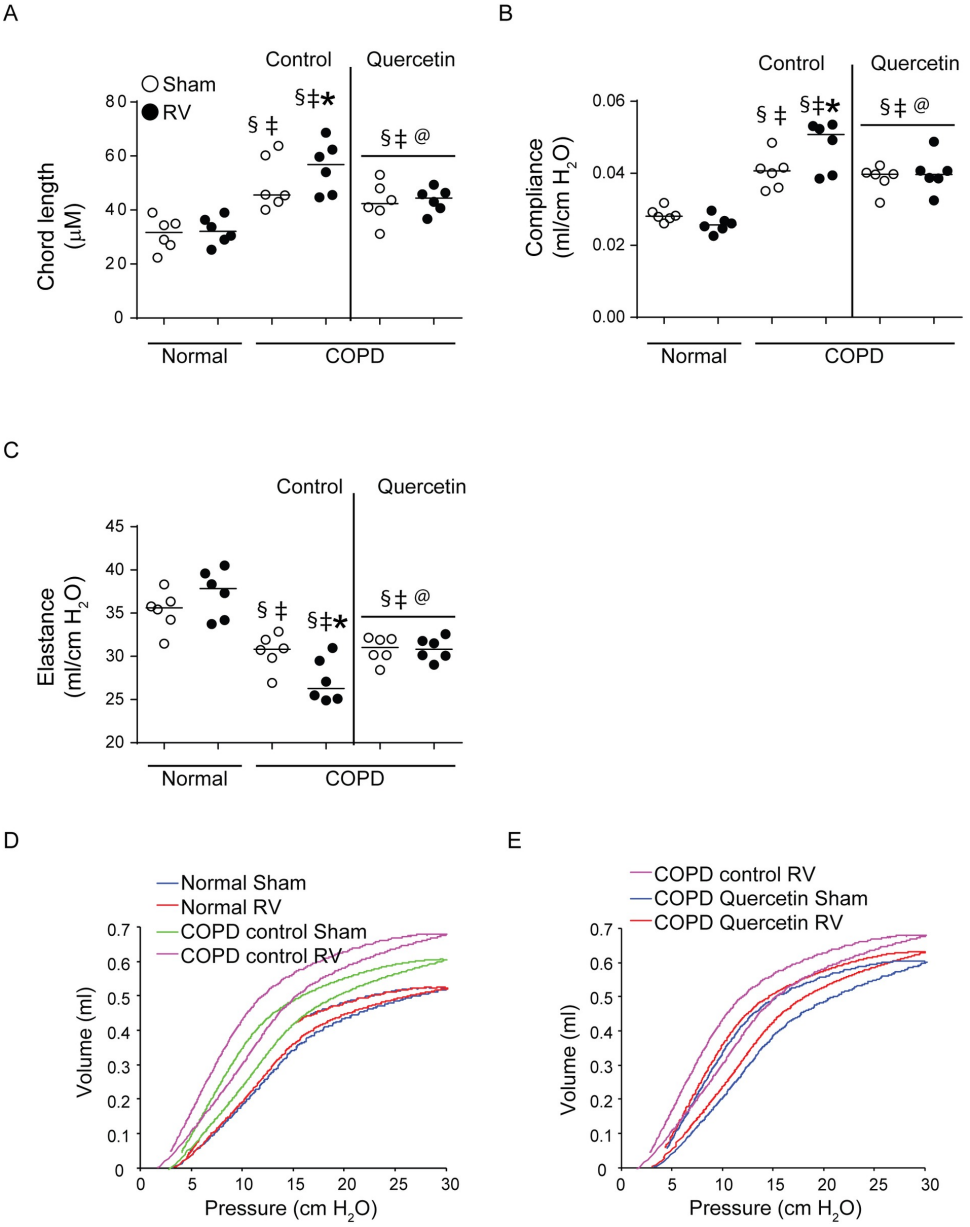
Quercetin abrogates progression of emphysematous changes in RV-infected mice with COPD phenotype. (A) Lung sections from sham- or infected mice with COPD phenotype maintained on control or quercetin diet and normal mice were stained with H & E and the diameters of the air spaces were measured in at least 10 random fields per section. (B to D) anesthetized mice intubated and connected to mechanical ventilator were used to measure dynamic compliance and elastance, and pressure-volume relationship. Data (A to C) represent median with range calculated from two independent experiments with 6 mice per group in total (* p≤0.05, different from respective sham; § p≤0.05, different from normal sham; ‡ p≤0.05, different from normal RV; @ p≤0.05, different from RV-infected COPD mice on control diet, ANOVA on ranks with Kruskal-Wallis H test). Data in (D and E) are representative of 6 mice per group.

Histological evaluation suggested that RV further enhances emphysematous changes in mice with COPD phenotype (Fig 2D). To quantify emphysematous changes we determined chord length, elastance, compliance and pressure volume loops. As previously observed mice with COPD phenotype showed increase in chord length (Fig 10A). This was accompanied by increase in dynamic compliance and decrease in elastance compared to normal mice (Fig 10B and 10C). Mice with COPD phenotype also showed left and upward shift in pressure-volume loops than normal mice, indicative of reduced elastic recoiling of the lungs as previously observed (Fig 10D). After RV infection, mice with COPD phenotype showed further increase in chord length and compliance, and decrease in elastance and elastic recoiling. These results corroborated with histological evaluation. Mice maintained on quercetin diet showed no change in either chord length, compliance or elastance, and elastic recoiling as indicated by pressure-volume loops (Fig 10E) following RV infection indicating quercetin blocks RV-induced progression of emphysematous changes in mice with COPD phenotype.

### RV-induced airway resistance is attenuated in quercetin-fed mice with COPD phenotype

**Fig 11:**
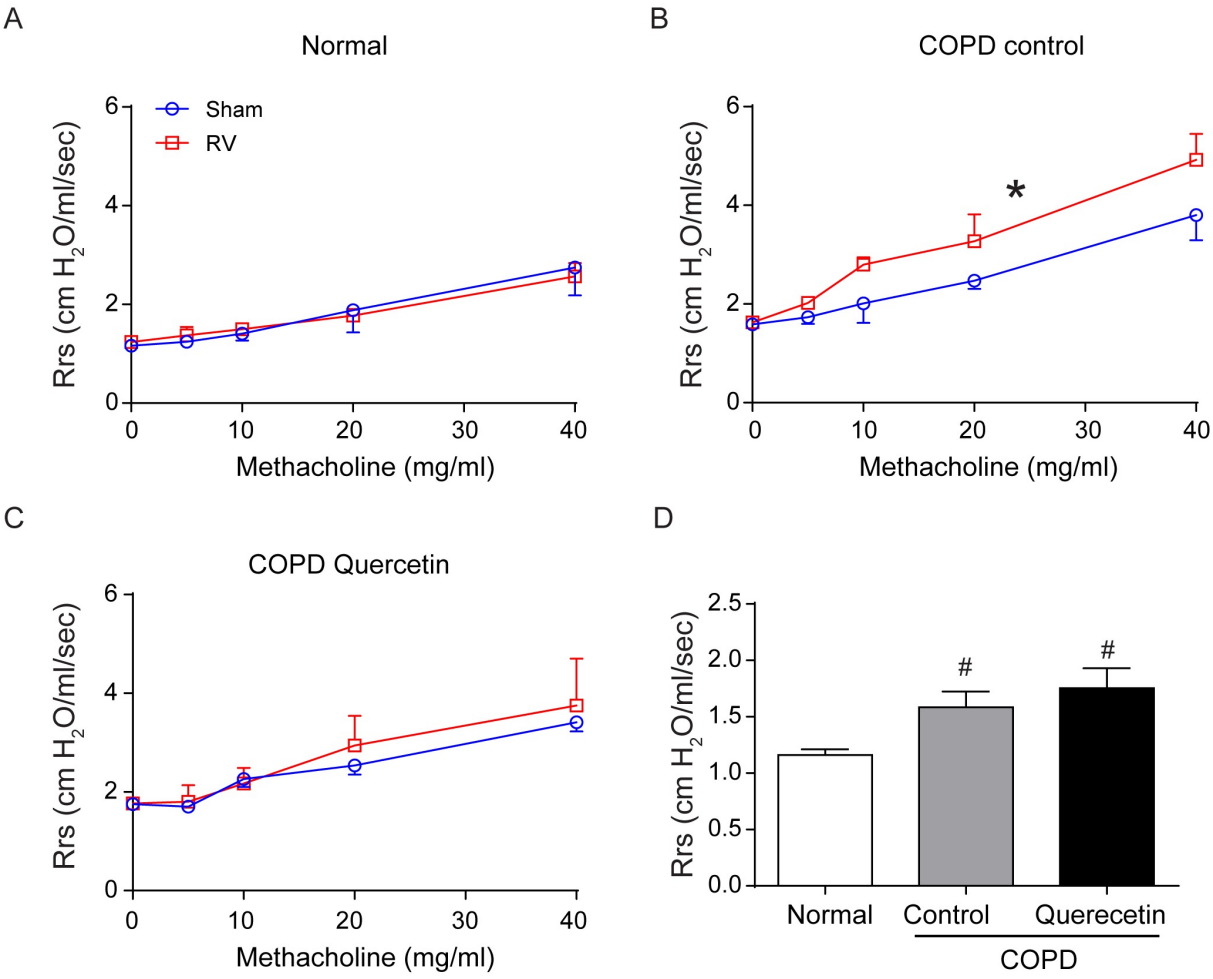
Quercetin inhibits RV-induced airway hyperreactivity in mice with COPD phenotype. (A and B) After relevant treatment, mice were anesthetized, intubated and connected to mechanical ventilator and airways responsiveness to nebulized increasing dose of methacholine was determined. (D) mice with COPD phenotype show higher airway resistance at baseline. Data represent mean and SEM calculated from 6 animals (* p≤0.05, different from respective sham, Two-way ANOVA; # p≤0.05, different from normal sham, ANOVA).

Airway cholinergic responsiveness was measured 14 days after RV infection. Normal mice infected with RV and sham showed very similar responsiveness to methacholine challenge (Fig 11A). In contrast, compared to sham-, RV-infected mice with COPD phenotype showed significantly higher response to methacholine challenge (Fig 11B). Mice on quercetin diet did not show such increase in airway responsiveness to methacholine challenge following RV infection (Fig 11C). Interestingly, we found that mice with COPD phenotype had higher basal airway resistance compared to normal mice (Fig 11D) and this may indicate airway constriction in these mice.

Taken together these results indicate that RV infection further increases lung inflammation and causes progression of lung disease including goblet cell metaplasia in the airways causing enhanced mucin gene expression, emphysema and airway function. Quercetin inhibits these RV-induced effects in mice with COPD phenotype.

## Discussion

This study provides first experimental evidence that RV causes persistent lung inflammation, mucus metaplasia and emphysematous changes up to 14 days in a relevant (smoke exposed) mouse model of COPD. Interestingly, this appears to be dependent on sustained host responses rather than viral persistence. Some of the pathologic features observed in this mouse model of COPD recapitulates RV-induced pathologic changes in experimentally-infected COPD patients with mild disease. These pathological features include a) persistent lung inflammation with accumulation of inflammatory cells including neutrophils, macrophages and T cells, and b) enhanced mucus production, airway resistance and progression of emphysema, which together may cause airflow obstruction [8]. Quercetin, a natural flavonoid with potent anti-inflammatory and antioxidant properties, abrogated RV-induced pathological changes in this mouse model of COPD.

In our previous study, we demonstrated that RV induces acute lung inflammation in both normal and mice with COPD phenotype [21]. While normal mice infected with RV resolved lung inflammation by 4 days, mice with COPD phenotype showed sustained lung inflammation. In the present study, we extended the time course up to 14 days to assess RV-induced lung inflammation and viral persistence. While RV-infected normal mice completely resolved lung inflammation at 14 days post-infection, mice with COPD phenotype showed enhanced peribronchiolar and perivascular inflammation. The observed persistent lung inflammation was not due to defective viral clearance, because there was no detectable viral RNA beyond 7 days post-infection in these mice. Differential cell counts indicated increased accumulation of neutrophils, macrophages, particularly intermediate macrophages, and CD8+ T cells in the lungs of mice with COPD phenotype. In contrast, normal mice showed only increase in CD8+ T cells, however it was significantly lower than in mice with COPD phenotype. Similar increase in T cells and neutrophils have been reported in COPD subjects who were experimentally infected with RV [8,9] indicating that this mouse model may be useful in obtaining mechanistic insight into RV-induced prolonged lung inflammation in COPD. Additionally, this experimental mouse model of COPD may also be useful for testing new therapeutic strategies to treat COPD exacerbations.

Persistent lung inflammation induced by RV in mice with COPD phenotype was associated with progression of lung disease encompassing both conductive airways and alveolar compartment. In the conductive airways, RV induced goblet cell metaplasia and mucin gene expression, one of the features of airway epithelial remodeling. This was not associated with increase in IL-13 expression (data not shown) indicating that pathways other than IL-13 may contribute to RV-induced goblet cell metaplasia. Our on-going studies indicate a role for NOTCH-dependent mechanism in RV-induced goblet cell metaplasia in COPD airway epithelial cells and this is yet to be confirmed in this mouse model of COPD. In alveolar compartment, RV causes enlargement of air spaces indicative of degradation of alveoli leading to progression of emphysema. This may be due to expression of MMP12 by lung macrophages, accumulation of which is increased in RV-infected mice with COPD phenotype. Moreover, previously we have demonstrated that RV induces MMP12 expression particularly in airway epithelial cells isolated from COPD patients [26]. Additionally, RV has also been shown to induce MMP9 expression in airway epithelial cells [27]. Furthermore, expression of MMP12 is increased in the lungs of COPD patients and has been implicated in pathogenesis of emphysema [28,29]. Consistent with this notion, MMP12 knockout mice are protected from developing emphysema [30]. Therefore, it is plausible that RV-induced MMP’s may cause progression of emphysema-like feature in mice with COPD phenotype.

Prolonged lung inflammation in RV-infected mice with COPD phenotype was associated with sustained expression of chemoattractants, CCL3 and CXCL-10, Th1 cytokines, TNF-α and IFN-γ, and Th17 cytokine IL-17A. Both CCL3 and CXCL-10 are potent chemoattractant for T cells and recruits T cells into tissues, which in turn may increase expression of TNF-α, IFN-γ and IL-17A. This is consistent with earlier report in which enhanced Th1 responses was observed in mice exposed to cigarette smoke and infected with influenza virus [31]. However, one cannot rule out the possibility of pulmonary macrophages contributing to the observed increases in CXCL-10 and TNF-α, because macrophages have been shown to express CXCL-10 and TNF-α in response to rhinovirus infection *in vitro* [32,33]. Currently, studies are underway in our laboratory to determine the source of these cytokines, and contribution of these cytokines in the recruitment and persistence of neutrophils, intermediate macrophages and CD8+ T cells.

Mice with COPD phenotype also showed increased airway responsiveness to cholinergic challenge following RV infection and this may be due to increase in the expression of TNF-α and CXCL-10. Both these cytokines have been shown to increase airways hyper responsiveness in mice [34,35]. However it remains to be confirmed whether these cytokines contribute to RV-induced airways hyper responsiveness to methacholine challenge in a mouse model of COPD.

Another important finding of this study is marked attenuation of RV-induced inflammatory changes and progression of lung disease in a mouse model of COPD by quercetin. Being a potent anti-oxidant and anti-inflammatory agent, quercetin may suppress persistent activation of epithelial cells induced by RV thus attenuating accumulation and activation of immune cells. Consistent with this notion, quercetin was recently shown to universally suppress accumulation and activation of immune cells and to improve mitochondrial function in the adipose tissue of diet-induced obese mice [36]. Mitochondrial dysfunction is one of the features of COPD [37]. Since mitochondria plays an important role in antiviral defenses and thus the outcome of viral infection, it is plausible that quercetin may alleviate RV-induced inflammatory changes by improving mitochondrial function and/or by directly inhibiting the viral replication. Previously, we and others have shown that quercetin inhibits respiratory viral replication both *in vitro* and *in vivo* by interfering at various stages of replication including viral endocytosis, viral genome transcription and translation [17,38]. In the present study, quercetin supplemented mice with COPD phenotype showed lower viral load than mice on control diet. Therefore, it is conceivable that quercetin may alleviate RV-induced pathogenic effects not only by limiting the host inflammatory responses to RV, but also by enhancing the viral clearance. Based on these facts we speculate that quercetin may mitigate RV-induced pathological changes and progression of lung disease in COPD following viral associated exacerbations. However, further experimental evidence is required prior to using quercetin as a new therapeutic strategy to prevent virus-induced exacerbations in COPD patients.

In conclusion, our findings demonstrate that RV induces lung inflammation with accumulation of neutrophils, T cells and macrophages in a mouse model of COPD. Mice with COPD phenotype also show progression of lung disease following RV infection and this was mitigated effectively by quercetin supplementation in the diet. In some aspects, this mouse model recapitulated the clinical outcome observed in COPD subjects, who were experimentally-infected with RV. Based on these observations, we conclude that the mouse model of COPD described here may be useful in providing mechanistic insights into RV-induced pathogenesis in COPD, and also for testing new therapeutic strategies. Secondly, quercetin supplementation with or without conventional steroid therapy may prevent severity of COPD exacerbations and progression of lung disease.

## Supporting information

S1 Fig Normal mice infected with RV do not show inflammation at 14 days post-infection. Normal mice infected with RV do not show inflammation at 14 days post-infection. Normal mice were infected with sham or RV by intranasal route and sacrificed 14 days after infection. Lungs were perfused with PBS, fixed and embedded in paraffin. Five micron thick paraffin sections were deparaffinized and stained with H & E and subjected to light microscopy. Images are representative of 6 mice per group.
(PDF)

S2 Fig Lung Viral RNA load in normal and mice with COPD phenotype. Lung Viral RNA load in normal and mice with COPD phenotype. Mice with COPD phenotype were shifted to control or quercetin diet. One week later mice with COPD phenotype and normal mice were infected with sham or RV and sacrificed at 2, 4, 7 and 10 days. Total RNA was isolated from the lungs and the viral RNA copy number was determined by quantitative qPCR and expressed as viral RNA copies/ 10 μg of total RNA. Experiment was conducted 2 times with 3 mice per group. Data represent median with range (*p≤0.05, different from normal mice; # p≤0.05, different from normal mice, ANOVA on Rank with Kruskal-Wallis H test).
(PDF)

S3 Fig Quercetin attenuates RV-induced cytokine responses. Quercetin attenuates RV-induced cytokine responses. Mice with COPD phenotype either maintained on control or quercetin diet were infected with sham or RV. Mice were sacrificed after 14 days, total lung RNA isolated and expression of cytokines were measured by qPCR. Data represent mean ± SEM calculated from 2 independent experiments performed with 2 to 4 mice per group/experiment (*p≤0.05, different from respective sham-infected animals; # p≤0.05, different from RV-infected mice on control diet, ANOVA with Tukey post-hoc analysis).
(PDF)
