# Supplementary material for: Quercetin prevents rhinovirus-induced progression of lung disease in mice with COPD phenotype
Source: PLoS One. 2018 Jul 5;13(7):e0199612. doi: 10.1371/journal.pone.0199612 (PMC6033397; doi:10.1371/journal.pone.0199612)
Supplement: S1 Fig — Normal mice infected with RV do not show inflammation at 14 days post-infection. Normal mice were infected with sham or RV by intranasal route and sacrificed 14 days after infection. Lungs were perfused with PBS, fixed and embedded in paraffin. Five micron thick paraffin sections were deparaffinized and stained with H & E and subjected to light microscopy. Images are representative of 6 mice per group. (PDF) [file pone.0199612.s001.pdf]

S1 Fig. Normal mice infected with RV do not show inflammation at 14 days post-infection

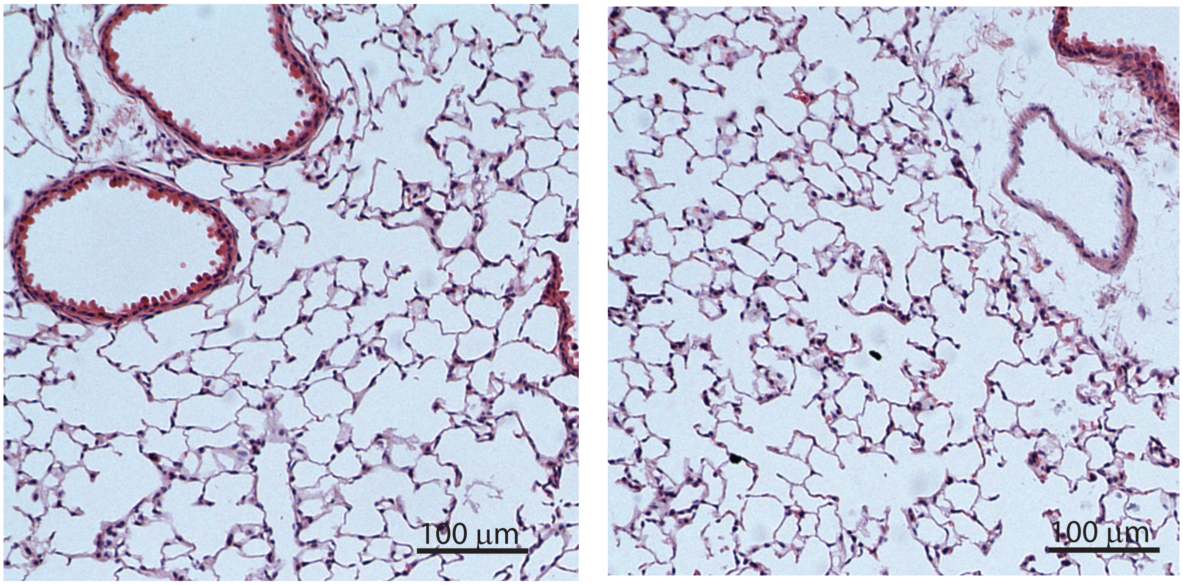

Sham RV

Normal mice infected with RV do not show inflammation at 14 days post-infection. Normal mice were infected with sham or RV by intranasal route and sacrificed 14 days after infection. Lungs were perfused with PBS, fixed and embedded in paraffin. Five micron thick paraffin sections were deparaffinized and stained with H & E and subjected to light microscopy. Images are representative of 6 mice per group.
