# Supplementary material for: Quercetin prevents rhinovirus-induced progression of lung disease in mice with COPD phenotype
Source: PLoS One. 2018 Jul 5;13(7):e0199612. doi: 10.1371/journal.pone.0199612 (PMC6033397; doi:10.1371/journal.pone.0199612)
Supplement: S2 Fig — Lung Viral RNA load in normal and mice with COPD phenotype. Mice with COPD phenotype were shifted to control or quercetin diet. One week later mice with COPD phenotype and normal mice were infected with sham or RV and sacrificed at 2, 4, 7 and 10 days. Total RNA was isolated from the lungs and the viral RNA copy number was determined by quantitative qPCR and expressed as viral RNA copies/ 10 μg of total RNA. Experiment was conducted 2 times with 3 mice per group. Data represent median with range (*p≤0.05, different from normal mice; # p≤0.05, different from normal mice, ANOVA on Rank with Kruskal-Wallis H test). (PDF) [file pone.0199612.s002.pdf]

S2 Fig. Lung Viral RNA load in normal and mice with COPD phenotype

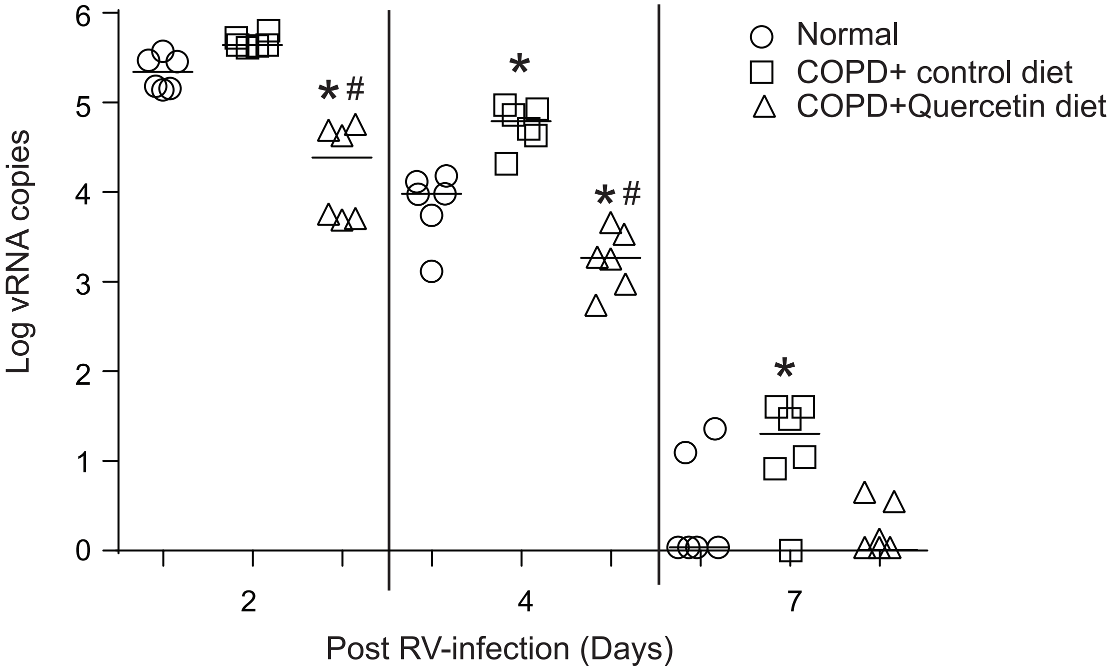

Lung Viral RNA load in normal and mice with COPD phenotype. Mice with COPD phenotype were shifted to control or quercetin diet. One week later mice with COPD phenotype and normal mice were infected with sham or RV and sacrificed at 2, 4, 7 and 10 days. Total RNA was isolated from the lungs and the viral RNA copy number was determined by quantitative qPCR and expressed as viral RNA copies/ 10 µg of total RNA. Experiment was conducted 2 times with 3 mice per group. Data represent median with range (*p≤0.05, different from normal mice; # p≤0.05, different from normal mice, ANOVA on Rank with Kruskal-Wallis H test).
