# Supplementary material for: Quercetin prevents rhinovirus-induced progression of lung disease in mice with COPD phenotype
Source: PLoS One. 2018 Jul 5;13(7):e0199612. doi: 10.1371/journal.pone.0199612 (PMC6033397; doi:10.1371/journal.pone.0199612)
Supplement: S3 Fig — Quercetin attenuates RV-induced cytokine responses. Mice with COPD phenotype either maintained on control or quercetin diet were infected with sham or RV. Mice were sacrificed after 14 days, total lung RNA isolated and expression of cytokines were measured by qPCR. Data represent mean ± SEM calculated from 2 independent experiments performed with 2 to 4 mice per group/experiment (*p≤0.05, different from respective sham-infected animals; # p≤0.05, different from RV-infected mice on control diet, ANOVA with Tukey post-hoc analysis). (PDF) [file pone.0199612.s003.pdf]

S3 Fig. Quercetin attenuates RV-induced cytokine responses

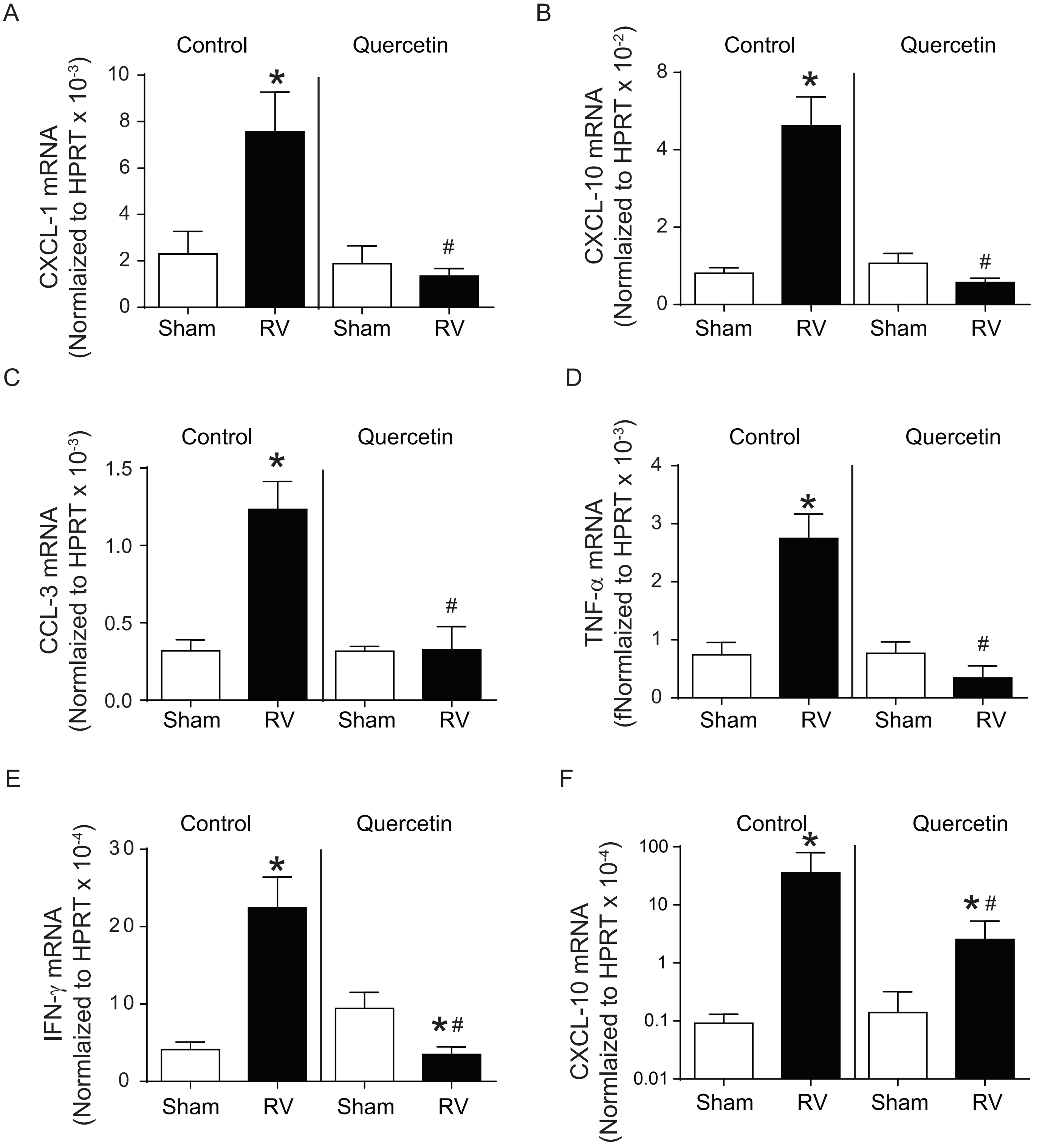

Quercetin attenuates RV-induced cytokine responses. Mice with COPD phenotype either maintained on control or quercetin diet were infected with sham or RV. Mice were sacrificed after 14 days, total lung RNA isolated and expression of cytokines were measured by qPCR. Data represent mean ± SEM calculated from 2 independent experiments performed with 2 to 4 mice per group/experiment (*$p \leq 0.05$, different from respective sham-infected animals; # $p \leq 0.05$, different from RV-infected mice on control diet, ANOVA with Tukey post-hoc analysis).
